# c-myc Regulates Cell Proliferation during Lens Development

**DOI:** 10.1371/journal.pone.0087182

**Published:** 2014-02-04

**Authors:** Gabriel R. Cavalheiro, Gabriel E. Matos-Rodrigues, Anielle L. Gomes, Paulo M. G. Rodrigues, Rodrigo A. P. Martins

**Affiliations:** Programa de Biologia Celular e do Desenvolvimento, Instituto de Ciências Biomédicas, Universidade Federal do Rio de Janeiro, Rio de Janeiro, Rio de Janeiro, Brazil; University of Dayton, United States of America

## Abstract

Myc protooncogenes play important roles in the regulation of cell proliferation, growth, differentiation and survival during development. In various developing organs, c-myc has been shown to control the expression of cell cycle regulators and its misregulated expression is detected in many human tumors. Here, we show that c-myc gene (Myc) is highly expressed in developing mouse lens. Targeted deletion of c-myc gene from head surface ectoderm dramatically impaired ocular organogenesis, resulting in severe microphtalmia, defective anterior segment development, formation of a lens stalk and/or aphakia. In particular, lenses lacking c-myc presented thinner epithelial cell layer and growth impairment that was detectable soon after its inactivation. Defective development of c-myc-null lens was not caused by increased cell death of lens progenitor cells. Instead, c-myc loss reduced cell proliferation, what was associated with an ectopic expression of Prox1 and p27^Kip1^ proteins within epithelial cells. Interestingly, a sharp decrease in the expression of the forkhead box transcription factor Foxe3 was also observed following c-myc inactivation. These data represent the first description of the physiological roles played by a Myc family member in mouse lens development. Our findings support the conclusion that c-myc regulates the proliferation of lens epithelial cells *in vivo* and may, directly or indirectly, modulate the expression of classical cell cycle regulators in developing mouse lens.

## Introduction

Complex developmental processes must be carefully orchestrated for the correct formation of the vertebrate eye. Even though optic-cup morphogenesis was recently reproduced *in vitro* by the use of three-dimensional stem cell culture systems [1], the molecular mechanisms driving eye organogenesis *in vivo* are still a major question in developmental biology. Concomitant with the coordinated growth of the developing lens and retina, the formation of a functionally mature eye depends on the precise coordination of cell proliferation, cell cycle exit and cell differentiation within these structures. In the past several years, a lot has been learned about the mechanisms that regulate these events, including the cell-extrinsic cues, such as growth factors, and cell-intrinsic factors, including cell cycle proteins and transcriptional regulators. Importantly, several homeodomain-containing proteins that act as transcription factors were identified and characterized as regulators of cell proliferation and cell differentiation in the developing lens. In contrast, few studies described the roles of basic-helix-loop-helix (bHLH) transcription factors in lens development [2]–[5]. Some recent studies addressed how these transcriptional networks functionally interact *in vivo* to regulate cell proliferation during lens ontogenesis [6], [7].

The refined architecture and well characterized stages of the developing vertebrate lens makes it an excellent model to study how these basic cellular processes are regulated in coordination. During embryonic development of the mouse, the contact between the optic vesicle and the head surface ectoderm occurs around embryonic day 9 (E9) and triggers the thickening and invagination of the ectoderm, forming the lens vesicle. Then, cells located on the posterior end of the vesicle exit cell cycle and elongate to terminally differentiate into primary lens fiber cells, while anterior cells continue proliferating and form the lens epithelia. At later stages, cell proliferation continues in the germinative zone from where cells migrate towards lens equator, exit cell cycle and start the secondary differentiation process. Through adulthood, epithelial cells exit the cell cycle and differentiate into fiber cells at the equatorial region of the lens. The adult lens is composed of post mitotic terminally differentiated fiber cells and a layer of cuboidal epithelial cells in its anterior region. Some of these epithelial cells remain as proliferative stem/progenitor cells [8]. Proper lens organogenesis requires the maintenance of the epithelial identity and cell proliferation by lens epithelial cells in precise coordination with cell cycle exit and cell differentiation. Many secreted growth factors, such as FGF, BMP and others [9]–[12], are known regulators of these events.

The molecular mechanisms driving cell proliferation in developing lens have been extensively studied [13]. Proper cell cycle exit and terminal differentiation of fiber cells critically depends on the Rb pathway. In Rb-null lens, cells in the transition zone fail to exit cell cycle [14]. A similar phenotype of hyperproliferation, followed by apoptotic cell death, was observed when CDK inhibitors p27^Kip1^ and p57^Kip2^ were both inactivated, suggesting that these CKIs functionally cooperate as upstream regulators of Rb pathway during lens terminal differentiation. These CDK inhibitors p27^Kip1^ and p57^Kip2^ are expressed in the fibers and their upregulation in the context of cell cycle exit depends on the transcription factor Prox1 (prospero-related homeobox 1) [15]. Regulation of cell cycle exit by Prox1 was previously shown in several developing tissues and in cancer [16]–[18] and evidence that Prox1 may regulate p27^Kip1^ transcription by directly binding to its promoter was also observed [19]. Little is known about how Prox1 expression is regulated during lens development [17]–[18], specially how its expression gets restricted to and maintained in early differentiating cells following terminal differentiation [20]–[22].

Previous studies have described that Myc transcription factors are expressed in developing lens of various vertebrates [23], [24]. The Myc family of proto-oncogenes includes c-myc (*Myc*), N-myc (*Mycn*) and L-myc (*Myc1*) that encode transcription factors containing basic-helix-loop-helix leucine zipper (bHLHZ) motifs and are known to regulate gene expression through a variety of mechanisms, including transcriptional activation through the formation of a heterodimer with Max as well as Max-independent mechanisms of c-myc-mediated transcriptional repression [25]–[28]. Myc proto-oncogenes have been shown to regulate cell survival, size, differentiation and specially cell proliferation in several developing organs, explaining why these transcription factors are absolutely crucial for life during development [27]. While N-myc plays a role in eye development by regulating retinal progenitor cells proliferation [29], previous studies, based in overexpression approaches, suggested that other Myc transcription factors may play a role in lens development [23], [30]. For instance, Ishibashi and colleagues reported the enlargement of the ocular globe as a result of c-myc overexpression driven by the Mx-promoter. However, the reported findings were either the result of overexpression of truncated c-myc in differentiating fiber cells [23] or overexpression was driven by promoters that increased c-myc expression in the cornea, iris, lens, and retina [30]. These nonspecific gain-of-function approaches made it impossible to clearly determine the tissue-specific roles of c-myc in eye development.

In the present study, we investigated whether c-myc regulates lens development *in vivo*. c-myc knockout mice die in uterus [31], so to determine the roles of c-myc in developing lens, we analyzed eyes and lenses of *Le-Cre; c-myc^flox/flox^* (*c-myc^Le-Cre^*) mice [32], [33]. The Le-Cre transgene is expressed in the surface ectoderm leading to inactivation of targeted alleles in both the lens and in the corneal epithelium [33]. Inactivation of c-myc by Le-Cre resulted in severe eye and lens growth impairment and anterior chamber malformation. In the absence of c-myc, no increase in cell death was detected and even though crystallin expression was normal, degeneration of fiber cells was observed at postnatal ages. For the first time we provide evidence that a sharp decrease in cell proliferation occurred after inactivation of c-myc. Consistently, ectopic expression of cell cycle exit proteins p27^Kip1^ and Prox1 was observed within epithelial cells and gene expression of Foxe3 and p27^Kip1^ was misregulated in c-myc-deficient lens.

## Materials and Methods

### Mice

Experimental procedures with animals were approved by the Committee of Ethics in Animal Use (CEUA) of the Health Science Center (CCS) based on the currently accepted international rules.

The c-myc floxed [32] (Myc^tm2Fwa^, MGI id:2178233) and the Lens-Cre [33] (Tg(Pax6-cre,GFP)1Pgr, MGI id:3045749) mice were previously generated and kindly shared. The control group (*c-myc^Ctrl^*) was composed of *c-myc* ^+/+^, *c-myc* ^+/F^ and *c-myc* ^F/F^. Mice with homozygous inactivation of c-myc specifically in the lens were identified as *c-myc^Le-Cre^*  =  *c-myc^F/F^*; Le-Cre^+/−^ and mice with heterozygosis of c-myc in the lens were identified as *c-myc^Het^*  =  *c-myc^+/F^*; Le-Cre^+/−^. To ensure that the offspring would inherit only one copy of the Cre transgene, Cre-positive animals (Le-Cre^+/−^ *; c-myc^F/F^*) were always mated to Cre-negative animals (Le-Cre^−/−^ *; c-myc^F/F^* or Le-Cre^−/−^ *; c-myc^+/F^*).

### RNA extraction, cDNA synthesis, and real-time RT-PCR analysis

Dissected lenses were obtained from staged embryonic (E12.5, E14.5, E17.5) and postnatal (P0, P3, P11, adult) C57BL/6 mice. RNA extraction and cDNA synthesis were performed as previously described [29], [34]. Real time RT-PCR reactions were performed in an ABI7500 machine (Applied Biosystems) using TaqMan® probes synthesized with 5′-FAM and 3′-BHQ for c-myc (*Myc*), Foxe3, p27^Kip1^ (*Cdkn1b*), actin (*Actb*) and GAPDH (*Gapd*). Primers used were listed in Table S1. Data analysis and normalization were performed as previously described [29], [34].

### Volume measurements of the eye and lens

The volume of postnatal (P0, P15) and adult (P30) eyes were measured as previously described [29]. After enucleation, eyes were fixed in phosphate-buffered saline (PBS)-buffered paraformaldehyde 4%. The axial length and two coronal axes (dorso-ventral and medial-lateral) of each eye were measured with a digital paquimeter and the eye volume was calculated after applying the formula (4/3× PI) × (eye axial length in mm) × (eye coronal length in mm) × (eye dorsal-ventral length in mm). After dissection of the retina and the lens, the same procedure was used to calculate lens volume.

### Histology and H&E staining

Embryos or eyes were collected in 4% PBS-buffered paraformaldehyde for 24 hours at 4°C and cryoprotected in 10, 20 and 30% (overnight) PBS-buffered sucrose solutions, respectively. Sections of 10 μm were obtained using a Leica 1850 cryostat. Hematoxilin (Proquimios) and Eosin (Merck) staining followed standard protocol.

### Immunohistochemistry

An antigen retrieval with citrate buffer was performed prior the antibodies incubation. The following antibodies were used: anti-PCNA (1∶500, Santa Cruz Biotechnology, cat#: sc-56), anti-Ser10 pH3 (1∶50, Cell Signaling, cat#: 9701), anti-active caspase-3 (1∶100, BD Biosciences, cat#: 559565), anti-Prox1 (1∶500, Covance, cat#: PR-238C), anti-p27Kip1 (1∶50, BD Biosciences, cat#: 610241), anti-cyclin D1 (1∶50, Cell Signaling, cat#: 2926), anti-ãH2AX (1∶300, Millipore, cat# 05-636), anti-E-cadherin (1∶100, Cell Signaling, cat#3195), anti-phopho-Erk1/2 (thr202/tyr204) (1∶500, Cell Signaling cat#: 4370). The á-crystallin (1∶50), â-crystallin (1∶300) and ã-crystallin (1∶50) antibodies were obtained from Dr. J. Samuel Zigler (Wilmer Eye Institute). The Foxe3 antibody (1∶150) was a gift from Dr. Peter Carlsson.

Immunohistochemistry reactions were developed with biotinilated secondary antibody (1∶400, Vector labs, cat#: BA2000 or BA1000) followed by ABC complex (Vector labs, cat#: PK6100) and DAB substrate kit (Vector labs, cat#: SK4100). Nuclear staining with methylgreen (Sigma Aldrich, cat#: 323829). Immunofluorescence reactions were developed by 2 alternative methods: biotinilated secondary antibody followed by ABC complex and Cy3-tyramide kit (Perkin Elmer, cat#: FP1046) or an Alexa 488 secondary antibody (1∶500, Life, cat#: A11001). Fluorescent nuclear counter staining were performed with Sytox Green (1/15000, Invitrogen, cat#: S7020) or with DAPI (Lonza, cat#: PA3013), respectively.

TUNEL analysis was performed following manufacturer instructions (Promega, cat#: G7362). Images were captured with a Leica TCS-SP5 with an AOBS system.

### Western blot

Protein extraction and blotting procedures were performed as previously reported [34]. Primary antibodies were as follows: c-myc (1∶1000, Cell Signaling, cat#: 5605), á-tubulin (1∶10000, Santa Cruz, cat #: sc32293). HRP-conjugated secondary antibodies from Cell Signaling (anti-mouse IgG, cat #: 7076, anti-rabbit IgG, cat #: 7074) were used at a 1∶1000 dilution. The ECL system (cat #: RPN2132) was used according to the manufacturer's instructions.

### Statistical analysis

t-test, one or two-way ANOVA were performed as indicated in the figure legends. p-values are based on two-sided tests. Tests were performed using Graphpad Prism software.

## Results

### Inactivation of c-myc in the surface ectoderm severely impairs lens and eye growth

**Figure 1:**
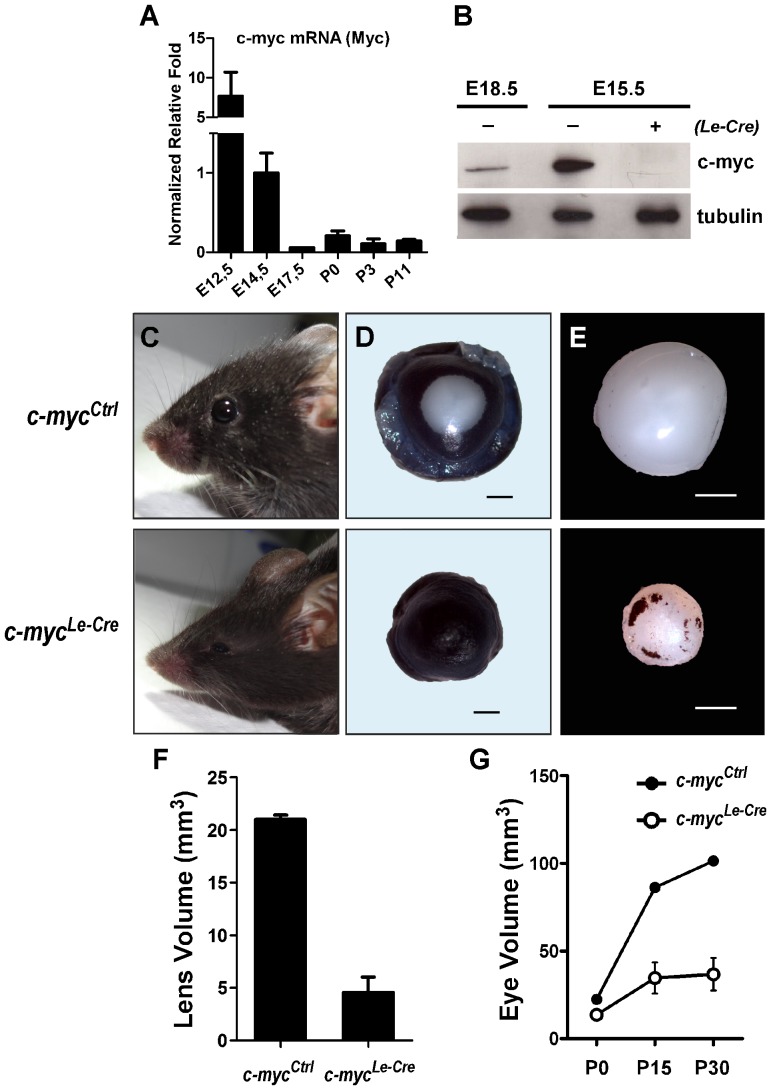
c-myc is highly expressed in developing mouse lens and genetic inactivation of c-myc in the surface ectoderm severely impairs lens and eye growth. (**A**) Real-time RT-PCR analysis of c-myc mRNA (*Myc*) expression at 6 stages of mouse lens development. Real-time RT-PCR data was obtained using TaqMan probes and datasets were normalized to actin (*Actb*). Normalized relative expression shows that *Myc* expression is highest in earliest stage analyzed (E12.5) and sharply decrease as development proceeds. (**B**) Western blot analysis of c-myc protein Expression (**C–E**) Representative pictures of *c-myc^Le-Cre^* and *c-myc^Ctrl^* mice (P60) (**C**), eyes (P15) (**D**) and lens (P30) at the indicated ages (**E**). (**F**) Measurement of lens volume at P30 shows that inactivation of c-myc in developing lens leads to a severe reduction of the lens volume (*c-myc^Le-Cre^*; n = 8; *c-myc^Ctrl^*; n = 6). (**G**) Inactivation of c-myc in the developing lens dramatically impairs eye growth as observed by the reduction of eye volume already at birth (P0; *c-myc^Le-Cre^*; n = 18; *c-myc^Ctrl^*; n = 14). Eye growth impairment was observed throughout postnatal development (P15; *c-myc^Le-Cre^*; n = 6; *c-myc^Ctrl^*; n = 4) and adulthood (P30; *c-myc^Le-Cre^*; n = 20; *c-myc^Ctrl^*; n = 12). A t-test was performed for (**F**) and a two-way ANOVA test was performed for (**G**). Error bars indicate SEM; *** p<0, 0001.

Previous studies described the expression of some Myc family transcription factors, including c-myc, in developing lens of different species [23], [24], [35]. To determine whether the relative amount of c-myc gene (*Myc*) expression would vary during mouse developing lens, we initially performed real time RT-PCR using c-myc-specific primers previously characterized [28] in various stages of lens development. c-myc mRNA expression was found as early as embryonic day 12.5 (E12.5), the earliest stage analyzed. Interestingly, c-myc gene expression sharply decreased thereafter remaining ∼8 to 100-fold smaller at all the older developmental stages analyzed (E14.5, E17.5, P0, P3, P11) (Figure 1A). We also measured c-myc protein expression in developing lens. In agreement with the gene expression data, western blot analysis of protein extracts from E15.5 and E18.5 lens showed that the amount of c-myc protein decreases during embryonic lens development (Figure 1B).

To analyze whether c-myc plays a role in mouse lens development, we generated *c-myc^Le-Cre^* (*c-myc^F/F^; Le-Cre^+/^* ^−^) mice in which c-myc was inactivated in the surface ectoderm. The Le-Cre transgenic mice present Cre recombinase activity in the lens placode as early as E9.5 [33]. Loss of c-myc protein in *c-myc^Le-Cre^* lenses was confirmed by western blot (Figure 1B). First, we asked whether inactivation of c-myc in the mouse lens would affect lens growth. At postnatal day 30 (P30), we observed a reduction of approximately 80% in lens volume (Figure 1C, D, E). The lens of *c-myc^Le-Cre^* mice were significantly smaller than *c-myc^Ctrl^* ones (21.01±0.42 mm^3^ vs. 4.58±1.44 mm^3^; p<0, 0001) (Figure 1F). In one of the *c-myc^Le-Cre^* mice no lens was formed at all, a phenotype known as aphakia. In the majority of *c-myc^Le-Cre^* mice we observed either a mild (up to 50% reduction in lens volume) or an aggressive lens volume reduction (reduction of more than 50% in lens volume) (Table 1). The severe lens growth defect observed did not allow us to perform precise measurements of the c-myc-deficient lenses at earlier stages of development.

**Table 1:** Eye phenotypes observed for control (*c-myc^Ctrl^*) and c-myc-deficient lens (*c-myc^Le-Cre^*).

Structure	Genotype	Stage	Total	Phenotype			
				None	Mild	Aggressive	Aphakia
***Eye***	*c-myc^Ctrl^*	P30	12	12	0	0	-
	*c-myc^Le-Cre^*	P30	20	0	0	20	-
	*c-myc^Ctrl^*	P15	4	4	0	0	-
	*c-myc^Le-Cre^*	P15	6	0	0	6	-
	*c-myc^Ctrl^*	P0	14	14	0	0	-
	*c-myc^Le-Cre^*	P0	18	0	6	12	-
***Lens***	*c-myc^Ctrl^*	P30	6	6	0	0	0
	*c-myc^Le-Cre^*	P30	8	0	2	4	2

Previous studies have demonstrated that eye growth during development depends on the correct development of the lens [9], [36]. As shown in figure 1 and in table 1, the impairment of lens development following c-myc inactivation also affected the growth of the whole eye. The eyes of *c-myc^Le-Cre^* mice were approximately 65% smaller during postnatal development (P15) and adulthood (P30) (Figure 1G). Notably, the eyes of mice with heterozygous lens (*c-myc^Het^*) were smaller then wild types, but significantly different from the homozygous (*c-myc^Le-Cre^*) P30 eyes (Figure S1). These findings suggest that the amount of c-myc protein within lens cells may be of functional relevance, since the phenotype observed may be correlated with c-myc content. Additionally, we observed that, at birth (P0), the eyes of *c-myc^Le-Cre^* mice were already smaller than the eyes of control littermates, suggesting that c-myc function is required during embryonic lens development (Figure 1G).

### Defective embryogenesis of the lens and anterior chamber

**Figure 2:**
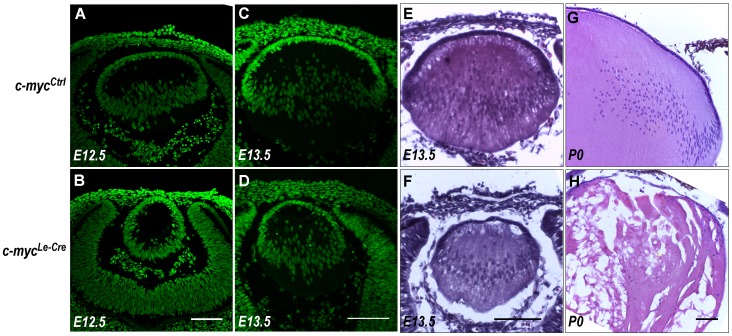
Defects in lens embryonic development of c-myc-deficient lens. **(A–D)** Representative confocal pictures of E12.5 (**A–B**) and E13.5 (**C–D**) control (*c-myc^Ctrl^*) and c-myc-deficient (*c-myc^Le-Cre^*) lens cryosections stained with sytox green. The pattern of nuclear staining of c-myc–deficient and control lens indicates a developmental delay. (**E–H**) Representative pictures of E13.5 sections of *c-myc^Le-Cre^* and *c-myc^Ctrl^* lens stained with hematoxilin and eosin (**E–F**) and P0 (**G–H**). At P0, the morphology of c-myc-null lens is severely compromised. Scale bar: 100 μm.

To characterize how c-myc-deficiency affects embryonic development of the lens, we first compared the morphology of *c-myc^Le-Cre^* and *c-myc^Ctrl^* lenses. At E12.5, the distribution of cell nuclei in control lens was characteristic of primary fibers cells that elongated from the posterior region of the lens vesicle. In contrast, c-myc-deficient lens displayed a vesicle-like morphology (Figure 2A–B). To determine whether the observed phenotype resulted from a developmental delay or from malformation we analyzed lens morphology at later stages. At E13.5, *c-myc^Le-Cre^* lens presented a distribution of cell nuclei (Figure 2C–D) and overall morphology (Figure 2E–F) characteristic of primary fibers cells elongating from posterior region of vesicle. At P0, a highly vacuolized fiber mass was observed in the lens of *c-myc^Le-Cre^* mice (Figure 2G–H). Altogether, these findings suggest that, in the absence of c-myc, a slight developmental delay occurs and that initial steps of fiber cell differentiation were not affected.

**Figure 3:**
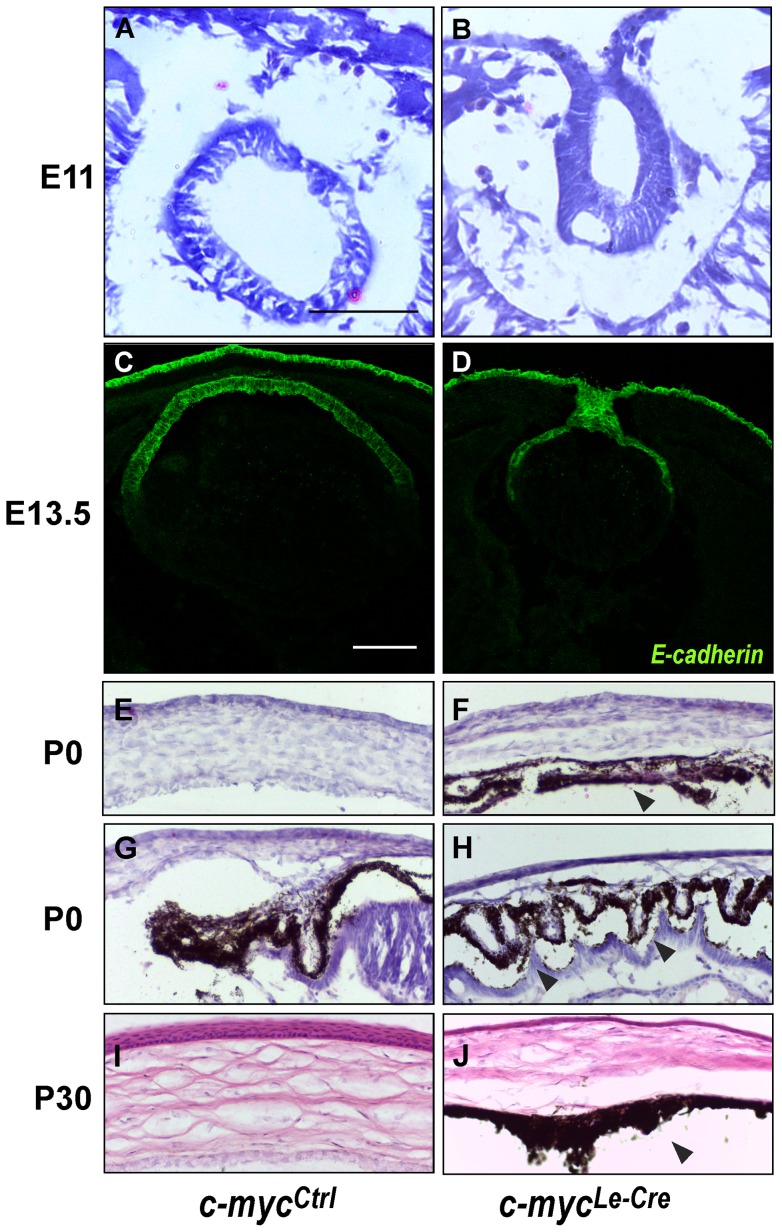
Loss of c-myc leads to development defects in the anterior chamber. Representative pictures of hematoxylin & eosin (H&E) staining at E11.0 (A, B) and immunofluorescence for E-cadherin at E13.5 (C, D) illustrates the defective lens vesicle formation, which leads to the formation of the lens stalk. (E–J) H&E staining of anterior segment at P0 (E–H) and P30 (I–J) demonstrates that *c-myc^Le-Cre^* eyes presented corneal stroma loosening, absence of corneal endothelium and presence of pigmented cells along the anterior chamber (at P0 and P30, arrowheads in F, H and J). Scale bar: 100 μm.

To evaluate whether the c-myc-deficiency affected earlier stages of lens development (e.g. lens vesicle formation), we performed H&E staining in sections of E11 eyes and counted the number of cells in the lens at this stage of development. No difference between *c-myc^Ctrl^* (62.67±5.510; n = 3) and *c-myc^Le-Cre^* (61.75±7.364; n = 4) was observed. However, a connection between the lens epithelium and the cornea (lens stalk) was observed in all animals analyzed at this stage (E11) and in more than 50% of the animals analyzed at E13.5 (Figure 3 A, B, C, D and Table 2).

**Table 2:** Phenotypes of control (*c-myc^Ctrl^*) and c-myc-deficient embryonic lens (*c-myc^Le-Cre^*).

Structure	Genotype	Stage	Total	Aphakia	Lens Stalk
***Lens***	*c-myc^Ctrl^*	E13.5	12	0	0
	*c-myc^Le-Cre^*	E13.5	9	0	5
	*c-myc^Ctrl^*	E11.0	5	0	0
	*c-myc^Le-Cre^*	E11.0	4	0	4

In addition, we observed that inactivation of c-myc in the surface ectoderm led to severe defects in the development of the anterior segment of *c-myc^Le-Cre^* mice. At P0, defects included corneal stroma loosening and absence of corneal endothelium. Furthermore, at P30, the *c-myc^Le-Cre^* mice presented thinner corneal epithelia. Other anterior segment structures were also affected. As shown in figure 3 (Figure 3E, F, G, H, I, J), it was not possible to distinguish the stroma of the ciliary body, the stroma of the iris or the chamber angle. In addition, we observed pigmented cells along the anterior segment (arrowheads in Figure 3).

### Inactivation of c-myc does not affect cell survival or early steps of lens fiber differentiation

**Figure 4:**
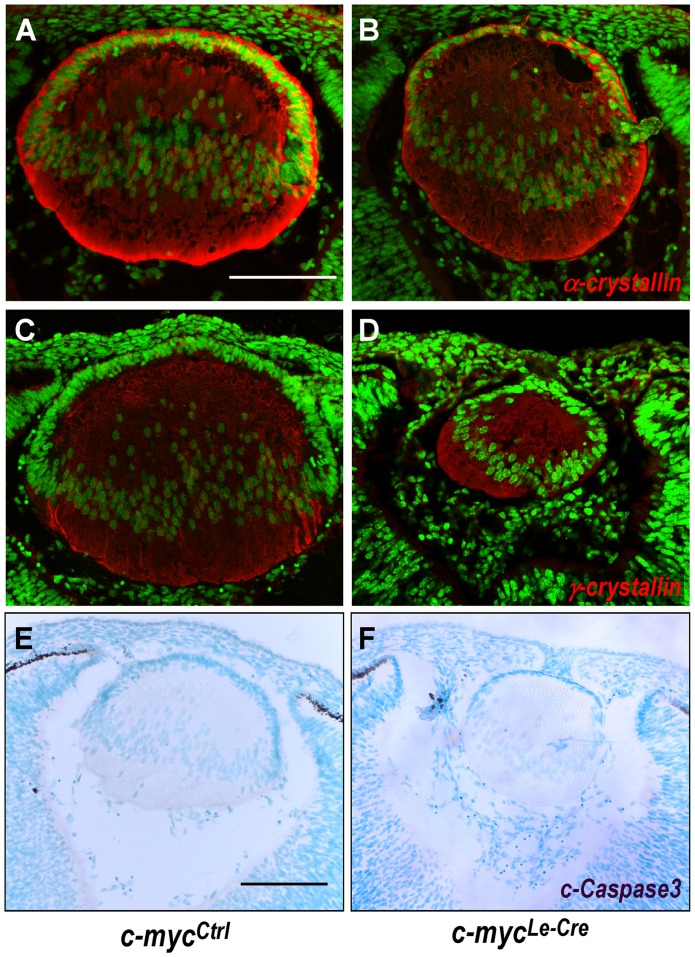
Inactivation of c-myc does not affect cell survival or the expression of crystallins. Representative confocal pictures of the immunofluorescence for á- crystallin (**A–B**) or ã-crystallin (**C–D**) performed in control (*c-myc^Ctrl^*) and c-myc-deficient (*c-myc^Le-Cre^*) lens sections at E13.5. Sytox nuclear counterstaining is shown in green. Immunostaining patterns are indistinguishable between c-myc–deficient and control lens. (**E–F**) Representative pictures of cleaved caspase-3 staining of control (**E**) and c-myc–deficient (F) lens at E13.5. c-myc loss did not lead to misregulation of apoptosis during embryonic lens development. Scale bar: 100 μm.

To test whether c-myc deficiency would lead to defects in cell differentiation during embryonic lens development, we analyzed classical aspects of fiber cells differentiation. First, we analyzed the pattern of á-, â- and ã-crystallins expression in c-myc-null lens, given the fact that their expression is a hallmark of appropriate fiber cell differentiation [9], [36]. At E13.5, no difference in the expression pattern of á- crystallin, â- crystallin or ã-crystallin was observed between *c-myc^Ctrl^* and *c-myc^Le-^* ^Cre^ (Figure 4A, B, C, D, and data not shown).

Several studies have demonstrated that signaling pathways activated by cell-extrinsic growth factors, such as FGF, lead to phosphorylation of ERK (pERK) and trigger fiber cell differentiation [37], [38]. Therefore, the pattern of Erk phosphorylation in embryonic lens may indicate whether fiber cells differentiation was correctly instructed and coordinated. No difference in pErk immunoreactivity was observed when E13.5 *c-myc^Le-Cre^* and *c-myc^Ctrl^* lenses were compared (Figure S2). These findings suggest that lens progenitor cells that became post-mitotic were able to normally start differentiation into fiber cells in the absence of c-myc.

Later in embryonic development, fiber cells initiate the terminal differentiation process, which depends on a controlled process of organelle degradation that will lead to the formation of the organelle free zone (OFZ) [39]. To analyze whether c-myc loss affects this event of terminal differentiation, we performed immunostaining for ãH2AX, a marker of nuclear degradation previously characterized in developing lens [40]. Even though, the histology of c-myc-deficient lens is already compromised at postnatal stages (Figure 2H), no difference in the pattern of ãH2AX when we compared *c-myc^Le-Cre^* and *c-myc^Ctrl^* lenses at birth. In addition, an organelle-free zone (OFZ) was detected in both control and c-myc-deficient lens at P0 (Figure S2).

As shown in figure 1, lens growth was severely compromised in c-myc-deficient lens. To directly analyze whether genetic inactivation of c-myc could lead to cell death in developing lens, we analyzed the proportion of apoptotic cells in both embryonic and postnatal *c-myc^Ctrl^* and *c-myc^Le-^* ^Cre^ lenses. At E13.5, no difference was observed in the proportion of cleaved caspase 3-positive cells (Figure 4E–F). Consistently, no change in the proportion of TUNEL-positive apoptotic cells was observed when we compared E12.5 *c-myc^Le-Cre^* and *c-myc^Ctrl^* lenses. Similar results were observed in postnatal (P0) lenses (Figure 2S).

### c-myc regulates epithelial cell proliferation in the developing lens

**Figure 5:**
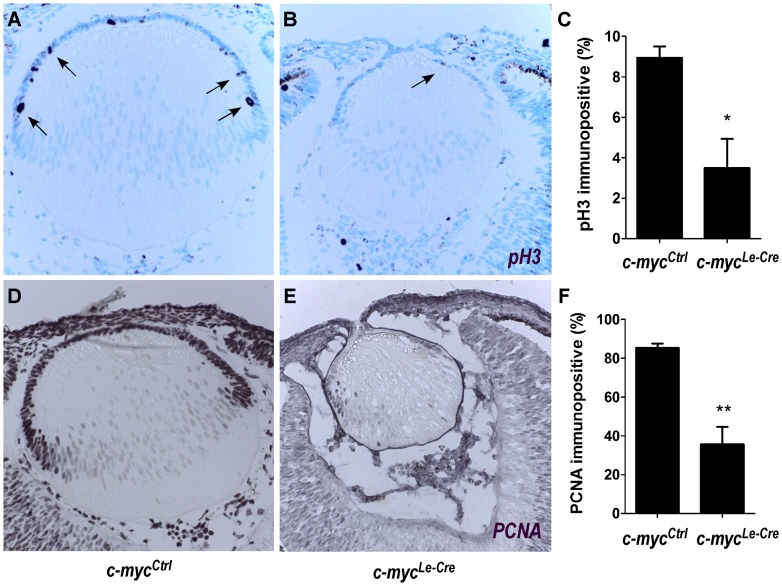
c-myc regulates lens epithelial cells proliferation. Immunohistochemistry for pH3 (**A–B**) and PCNA (**D–E**) were performed in control (*c-myc^Ctrl^*) and c-myc-deficient (*c-myc^Le-Cre^*) lens sections at E13.5 followed by methylgreen nuclear counterstaining. Proportions of pH3 immunopositive cells (arrows) (**C**) and PCNA immunopositive cells (**F**) were scored within lens epithelial cells (*c-myc^Le-Cre^*; n = 5; *c-myc^Ctrl^*; n = 4). Inactivation of c-myc reduced the proportion of proliferating cells within lens tissue *in vivo*. Error bars indicate SEM. A t-test resulted in * p<0, 05; ** p<0, 01.

Previous studies based on the overexpression of distinct Myc genes suggested c-myc could play a role in the regulation of cell proliferation in developing lens [23]. To test whether the proliferation of lens progenitor cells would be affected *in vivo* by c-myc loss, we first scored the proportion of phospho-histone H3 (pH3) immunopositive cells in *c-myc^Ctrl^* and *c-myc^Le-Cre^* lenses. At E13.5, we observed a reduction of approximately 60% in the proportion of pH3 positive cells in the absence of c-myc (Figure 5A, B, C). To provide further evidence of decreased cell proliferation, we quantified the proportion of PCNA immunopositive cells and similar results were observed. A significant reduction in the proportion of PCNA positive cells was observed in the lens vesicle (E11.5) (79.5±3.4% vs. 65.5±2.2%; p<0.05). Later, at E13.5, significantly fewer PCNA-positive cells were observed in the epithelia of the *c-myc^Le-Cre^* lens in comparison to the *c-myc^Ctrl^* ones (85.2±2.2% vs. 35.6±9.0%; p<0, 01) (data not shown and Figure 5D, E, F). Together, the pH3 and PCNA data suggest that c-myc regulate cell proliferation of lens progenitor cells as early as the vesicle stage.

### Misregulated expression of Prox1 and p27^Kip1^ proteins in c-myc-deficient lens

**Figure 6:**
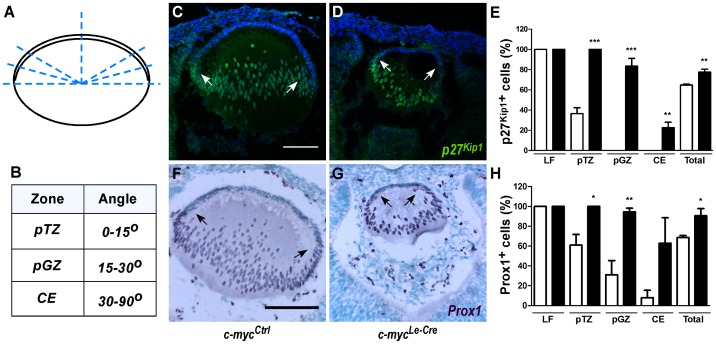
Misregulation of p27^Kip1^ and Prox1 proteins in c-myc-deficient lens. (**A–B**) Schematic illustration of the division of the lens cells in 4 regions that were independently quantified in stained sections: central epithelia (CE), prospective germinative zone (pGZ), prospective transition zone (pTZ) and lens fibers (LF). (**C–D**) Immunofluorescence for p27^Kip1^ and (**F–G**) immunohistochemistry for Prox1 were performed in control (*c-myc^Ctrl^*) and c-myc-deficient (*c-myc^Le-Cre^*) lens sections at E13.5 followed by nuclear either counterstaining with DAPI (**C–D**) or methylgreen (**F–G**), respectively. Proportions of p27^Kip1^ immunopositive cells (white arrows) and Prox1 immunopositive cells (black arrows) were scored in each of the regions CE, GZ, TZ and LF (*c-myc^Le-Cre^*; n = 3; *c-myc^Ctrl^*; n = 4). Inactivation of c-myc increased the proportion of cells expressing p27^Kip1^ and Prox1 within lens tissue *in vivo*. Error bars indicate SEM; * p<0, 05; ** p<0, 01; *** p<0, 001. Scale bar: 100 μm.

Appropriate timing of cell cycle exit is essential for proper cell differentiation during lens embryogenesis [13]. The reduction in cell proliferation following c-myc loss led us to test whether known regulators of lens cell cycle exit, such as Prox1 and p27^Kip1^ [15], [41] would be misregulated in the lens of *c-myc^Le-Cre^* mice. It's well characterized that the behavior of proliferating cells depends on the region of the epithelia occupied by these lens progenitors. Therefore, after staining sections of E13.5 *c-myc^Ctrl^* and *c-myc^Le-Cre^* lenses for p27^Kip1^ and Prox1, we performed a careful regionalized quantification of the immunopositive cells [42] to characterize whether alterations in the expression of these proteins would be specific for different zones of the lens epithelia (Figure 6A–B). As demonstrated in figures 6C, D, E, in the absence of c-myc, the proportion of p27^Kip1^–positive cells significantly increased in the prospective germinative zone (GZ), prospective transition zone (TZ) and in the central epithelia (CE) of the *c-myc^Le-Cre^* lenses (Figure 6E). Similar analysis was performed for Prox1 expression (Figure 6F, G, H). As observed for p27^Kip1^, the total proportion of Prox1-positive cells significantly increased in c-myc-deficient lens. Interestingly, the regions of Prox1 upregulation were slightly different from p27^Kip1^, since more Prox1-positive cells were detected in the prospective GZ and the prospective TZ, but not in the CE (Figure 6F).

**Figure 7:**
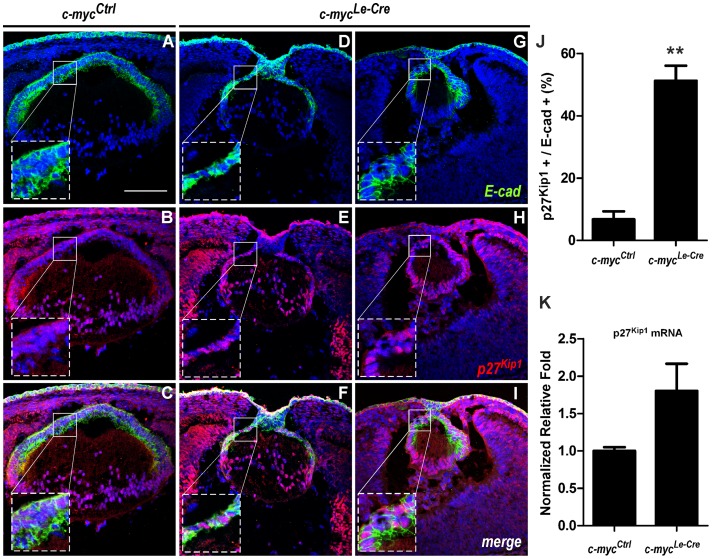
c-myc loss leads to ectopic expression of p27^kip1^ in the lens anterior epithelium. Representative confocal pictures of a double immunofluorescence for E-cadherin (**A, D, G**) (green), p27^kip1^ (**B, E, H**) (red) in control (*c-myc^Ctrl^*) and two different c-myc-deficient (*c-myc^Le-Cre^*) lens at E13.5. DAPI nuclear counterstaining is shown in blue. The insets show the IHC for p27^kip1^ and E-cadherin specifically in the lens anterior epithelium. (**J**) The proportion of p27^kip1+^ and E-cadherin^+^ cells increased in the *c-myc^Le-Cre^*. (**K**) Real-time RT-PCR analysis of p27^kip1^ mRNA (*Cdkn2b*) expression in control (*c-myc^Ctrl^*) and c-myc-deficient (*c-myc^Le-Cre^*) lens at E17.5. Gapdh (*Gapd*) was used to normalize p27^kip1^ mRNA (*Cdkn1b*) expression. Error bars indicate SEM; ** p<0, 01; Scale bar: 100 μm.

To provide further evidence that c-myc inactivation caused ectopic expression of p27^Kip1^ protein within lens epithelial cells, we performed a double immunostaining for E-cadherin, a classical marker of lens epithelial cells, and p27^Kip1^ in *c-myc^Ctrl^* and *c-myc^Le-Cre^* cryosections (Figure 7A, B, C, D, E, F, G, H, I). As observed in figures 7F and 7I, in the *c-myc^Le-Cre^* mice, E-cadherin positive cells located in the central epithelium were also expressing p27^Kip1^ protein. We quantified the proportion of double positive cells and verified that the proportion of epithelial cells expressing p27^Kip1^ protein increased ∼6 fold in c-myc-deficient lens. To quantify the mRNA expression of p27^Kip1^, we performed realtime RT-PCR in E17.5 lenses. A subtle increase (∼1.7 fold) in p27^Kip1^ gene expression was observed in c-myc-null lenses (Figure 7K).

**Figure 8:**
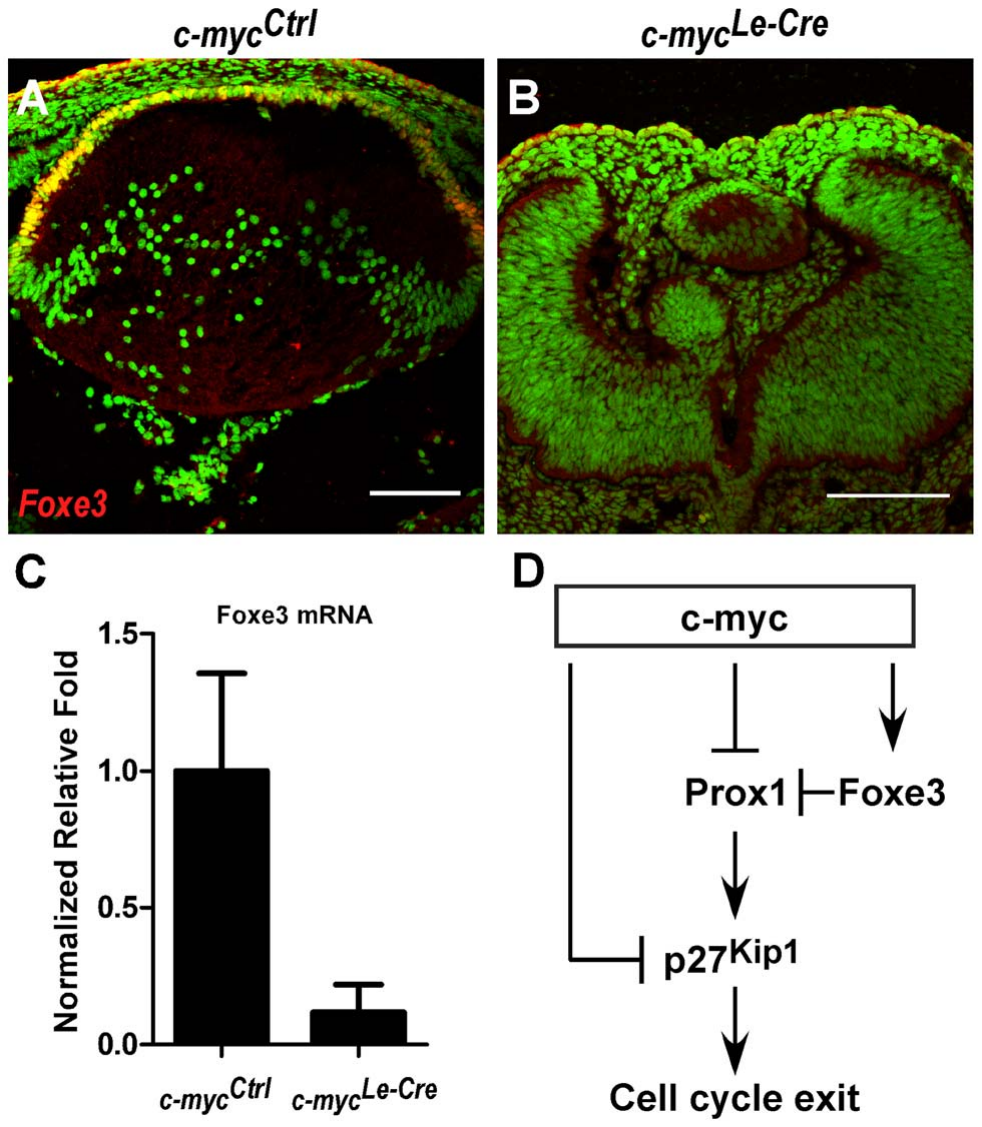
Misregulation of Foxe3 expression in c-myc-deficient lens. **(A–B)** Representative confocal pictures of an immunofluorescence for Foxe3 performed in control (*c-myc^Ctrl^*) (**A**) and c-myc-deficient (*c-myc^Le-Cre^*) (**B**) lens sections at E13.5 followed by sytox green nuclear counterstaining. (**C**) Real-time RT-PCR analysis of Foxe3 mRNA expression in control (*c-myc^Ctrl^*) and c-myc-deficient (*c-myc^Le-Cre^*) lens at E17.5. Gapdh (*Gapd*) was used to normalize Foxe3 expression. A sharp decrease in Foxe3 mRNA and protein expression was detected after c-myc inactivation (**D**) Schematic illustration of the proposed roles of c-myc in developing mouse lens. The ectopic expression of Prox1 and p27^Kip1^ proteins in lens epithelial cells of the *c-myc^Le-Cre^* mice suggests that the expression of these cell-cycle exit inducers may be regulated by c-myc in the embryonic lens. The expression of Foxe3, a known regulator of Prox1, was also misregulated in c-myc-deficient lens.

The observed phenotypes: _(1)_ ectopic expression of cycle exit regulators within epithelial cells and _(2)_ defective morphogenesis of the anterior chamber have some similarities with the ones observed in Foxe3-null mice [43]. To test whether the expression of Foxe3 would be misregulated in c-myc-deficient lens, we performed immunofluorescence to Foxe3. Interestingly, the expression of Foxe3 protein in the anterior epithelia of E13.5 *c-myc^Le-Cre^* mice was drastically reduced (Figure 8A–B). In addition, realtime RT-PCR analysis of E17.5 lens also demonstrated a decrease in Foxe3 gene expression (Figure 8C), suggesting that c-myc may regulate the expression of Foxe3 in developing lens. Altogether, our findings indicate, for the first time, that *in vivo* inactivation of c-myc in the surface ectoderm leads to defective development of the anterior segment and the lens, resulting in severe microphtalmia. We propose that misregulation of cell proliferation in developing lens contributes to the described phenotypes.

## Discussion

In this study, we found several lines of evidence that the proto-oncogene c-myc is required for proper development of the lens *in vivo*. First, we performed gene expression studies to confirm that this member of the bHLHZ transcription factor family is expressed during embryonic and postnatal development of the mouse lens. Importantly, we show that the amount of c-myc (*Myc*) transcripts sharply decreases during lens embryonic development and remain at lower levels from E17.5 through adulthood. Using genetic assays, we inactivated c-myc expression in a tissue-specific manner and demonstrated that the loss of c-myc, starting at the surface ectoderm stage, severely impairs lens and eye organogenesis. Consistent with previous studies that used alternative genetic approaches to alter c-myc expression in developing lens [23], we found no evidence for a role of c-myc in the regulation of cell survival in developing mouse lens. Even though we did not observe alterations in the expression pattern of crystallins and phosphorylated Erk during embryonic development or ãH2AX at early postnatal stages, degeneration of fiber cells in c-myc-deficient lens was observed at later stages of development. More importantly, we found that, in the absence of c-myc, cell proliferation was greatly reduced during embryonic development of the lens. In addition, we provided some evidences of the mechanisms of cell cycle control by c-myc in developing lens. Prox1 and p27^Kip1^ proteins are ectopically expressed in lens epithelial cells and a slight increase in p27^Kip1^ mRNA expression was observed in c-myc-null lens. Based on these findings, we propose that c-myc plays an important role in lens development through the regulation of the cell cycle in the lens progenitor cells. These data led us to propose the hypothetical model in which c-myc negatively regulates the expression of Prox1 and p27^Kip1^ in lens epithelial cells preventing lens progenitor cell cycle exit (Figure 8D).

### Regulation of cell proliferation, but not cell death, in lens development

Germ line inactivation of c-myc made it clear that c-myc was essential to life, because homozygous mice did not survive beyond E10.5 days of gestation [31]. In this study, no detailed description about the defects in eye development was provided. Even though it would have been possible to detect specific abnormalities in the formation of the lens vesicle, it was only briefly mentioned that optic development scored poorly in c-myc homozygous mice. In this context, our work contributes as the first example of a loss-of-function approach that clearly demonstrates a physiological role of a Myc family member in eye development.

Our data provide genetic evidence that c-myc is required for lens and eye development. As demonstrated in Figure 1 and Figure S1, eye and lens growth were severely compromised in *c-myc^Le-^* ^Cre^ mice. Notably, the reduction in adult eye volume was smaller for mice with one functional copy of c-myc. This observation that c-myc heterozygosis resulted in an intermediary phenotype suggests that the amount of this Myc protein may be of relevance for c-myc-mediated functions in developing lens, as well as undermines the possibility of a toxic role of Cre recombinase in the generation of the observed lens phenotypes. Importantly, eye volume was significantly smaller at birth, suggesting that c-myc function is required early in lens embryogenesis. Developmental defects in c-myc-deficient lens were observed as soon as E11.5 (Figure 3), approximately, 2 days after Cre mediated recombination is detected in the surface ectoderm [33]. The vesicle morphology of E12.5 c-myc-deficient lens showed that primary fiber cell differentiation was not yet initiated at this stage. Genetic inactivation of c-Maf proto-oncogene [44], [45] arrests lens development at the vesicle stage. To distinguish between such a severe malformation or a developmental delay in embryonic development, we analyzed the morphology of E13.5 lens and found evidence that primary fiber cell differentiation was normally initiated in the absence of c-myc (Figure 2). These findings suggested that the impaired organogenesis of *c-myc^Le-^* ^Cre^ eye and lens and eye were not caused by a complete impairment of lens vesicle formation or defects in fiber cell differentiation.

Inactivation of c-myc in embryonic lens tissue reduced the proportion of classical proliferation markers. The proportion of mitotic cells, stained for the phosphorylated form of histone H3 (pH3) was reduced in 60% (Figure 5). Since the mitotic cells are inevitably the smallest population of proliferating cells, we expanded the characterization of cell proliferation in c-myc-null lens analyzing another marker of this event (PCNA). Consistently, a ∼2.5-fold reduction in the proportion of PCNA-positive cells was detected within the lens tissue at E13.5. A similar, but less pronounced, decrease in the proportion of PCNA positive cells was detected at the lens vesicle stage (E11.5). These results suggest that the loss of c-myc function resulted in misregulation of the cell cycle of lens progenitor cells few days after its genetic inactivation.

In accordance with our findings, overexpression of c-myc in (driven by áA crystallin promoter) induced lens cells to enter the S-phase of the cell cycle [23]. Since, forced expression of c-myc resulted in cell cycle progression, it was suggested that c-myc may be sufficient to induce cells to re-enter cell cycle. Our work, add to the previous findings by showing that lens progenitor cells are able to proliferate in the absence of c-myc. It remains to be determined whether redundant or compensatory expression of another Myc gene in any of the proliferative cell populations of the lens is related with the maintenance of proliferation after c-myc loss. The possibility that c-myc expression and function is heterogeneous within subpopulations of lens progenitor may not be discarded.

Altogether, these findings strongly suggest that the impairment of lens and eye development here described is mainly caused by misregulation of cell proliferation following c-myc loss in developing lens.

As mentioned, c-myc inactivation in developing lens severely impaired eye and lens development (Figures 1 and 2). It has become clear that the decision of a cell to undergo apoptosis and the participation of c-myc in this process are specific for the cell type and biological context [46]. Therefore, we analyzed whether an increase in cell death could contribute to the phenotype discovered. The lack of c-myc protein in developing lens did not affected cell death in any of the stages studied, as verified by both TUNEL assay and staining for activated caspase 3 during embryonic and postnatal development (Figure 4 and Figure S2). It's still not determined whether c-myc-deficiency may lead to cell death at later stages of lens postnatal development (after birth). Consistent with our observations that c-myc genetic inactivation did not altered cell death in developing lens, when c-myc was overexpressed no increase in apoptosis was reported [23].

Even though, we favor the hypothesis that c-myc does not regulate cell death in developing lens, a role for c-myc in cell death during early steps of lens embryogenesis may not be completely discarded, since other Myc family members could compensate for c-myc loss.

### Molecular mechanisms regulated by c-myc in developing lens

In developing lens, Prox1 protein is first detected around E10.5 in cells of the anterior and posterior compartments of the lens vesicle. Following primary differentiation, at E12.5, Prox1 levels are still high in the nucleus of elongating fiber cells and decrease in lens epithelium. Afterwards, Prox1 protein expression becomes restricted to early differentiating cells [22]. To our knowledge c-myc has not been previously shown to regulate Prox1 expression.

Ectopic expression of Prox1 was demonstrated in Foxe3-null lens [43]. As shown in figure 8, we observed that the expression of Foxe3 protein is downregulated in c-myc-deficient lens epithelia. This effect is likely caused by a decrease in Foxe3 gene expression, since Foxe3 mRNA content was also reduced in *c-myc^Le-Cre^* lens (Figure 8). Therefore, it is reasonable that the ectopic expression of Prox1 we observed in c-myc-deficient lens is a consequence of Foxe3 downregulation. Alternatively, Prox1 transcription may directly or indirectly regulated by c-myc in a Foxe3-independent manner. It has been shown that the Myc-associated zinc finger protein (MAZ) may regulate the gene expression of Prox1 in hepatocellular carcinoma [47]. In addition, Pitx3 has been shown to play a role in the cell cycle of lens epithelial cells and fiber cell differentiation by positively regulating Foxe3 expression and negatively regulating Prox1 in the anterior lens epithelium. These effects culminated in prevention of p27^Kip1^ and p57^Kip2^ activation and maintenance of lens epithelial cells in cell cycle [20]. Therefore, it is possible that the regulation of p27^Kip1^ and/or Prox1 expression by c-myc depends on interactions with Foxe3 (Figure 8D) or Pitx3.

Binding of c-myc to initiator (Inr) elements can abolish Miz-1-mediated transcriptional activation. Repression of p27^Kip1^ expression following c-myc binding to Inr elements located in p27^Kip1^ promoter has been previously demonstrated [48]. It's known, however, that Prox1 regulates p27^Kip1^ in the lens [15], so it is possible that regulation of p27^Kip1^ expression by c-myc may be indirectly mediated by Prox1. Interestingly, our data supports previous findings [11], [22] that Prox1 and p27^Kip1^ are not always expressed in the same cells at the same developmental stage. Therefore, the c-myc-Prox1-p27^Kip1^ axis proposed in Figure 8D may not be found in every differentiating lens cell. Future studies are necessary to better determine the transcriptional network regulated by c-myc in developing lens.

### c-myc and anterior segment morphogenesis

The Le-Cre transgene leads to genetic inactivation in the surface ectoderm, that will give rise to the lens, and in the ocular surface epithelia (corneal, conjunctival and eyelid epithelia) [33], [57]. In addition to the lens phenotypes described above, we observed several defects in other structures of the anterior chamber following inactivation of c-myc from the surface ectoderm. In addition, we observed a remnant connection between the lens and the surface ectoderm – usually referred as lens stalk – as early as E11.5. Published studies report the degeneration of this connection between developing lens and cornea at slightly different stages. While some have described the presence of lens stalk in wild-type mice as late as E12–E12.5 [5], [49], [50], several other studies reported that this structure is already absent at E11.5 [51]–[53]. In our hands, no lens stalk was observed in control mice at ∼E11, but, in *c-myc^Le-Cre^* mice, the lens stalk was observed in all animals we analyzed (n = 4, Table 2). Few days later, (E13.5), more than 50% of the c-myc-deficient lens had remnant connection. These findings indicate that c-myc is necessary for proper lens vesicle separation from the surface ectoderm.

Anterior segment dysgenesis has been described in several human diseases (OMIM 107250), but relatively few transcription factors were shown to be critical for the development of the anterior segment of the eye in both human and mice [54]. Here, we reported that c-myc function is necessary for proper anterior segment morphogenesis. Around birth, a reduction in corneal thickness and corneal stroma loosening were detected in *c-myc^Le-Cre^* mice. In addition, it was not possible to distinguish the stroma of the ciliary body and of the iris and pigmented cells were detected along the anterior segment (Figure 3). It's clear that signals from the lens epithelium are required for proper differentiation of the cells that form the corneal endothelium, iris stroma and anterior chamber angle. For example, repositioning of the lens, in way that it does not face the anterior chamber, leads to defective anterior segment development [55]. It's also established that neural crest-derived mesenchymal cells contribute to the proper development of iris, ciliary process, corneal stroma and endothelium. Therefore, it's possible that the phenotypes here described are caused by defective cell differentiation in the iris/anterior segment and of these migrating mesenchymal cells that do not differentiate and end up located in the anterior chamber of the *c-myc^Le-Cre^* mice eyes.

Similar phenotypes have been shown in other transgenic mice that show defects in the lens epithelium [43], [56], [57]. In particular, anterior segment malformation is also found in *dyl* mice (Foxe3 mutated) [58]. As mentioned, we observed that the expression of Foxe3 mRNA and protein are downregulated in c-myc-deficient lens (Figure 8). It's reasonable to suggest that the anterior segment defects caused by c-myc loss are due to a decrease in the expression of Foxe3 in the lens epithelia. However, the possibility that c-myc may have cell-autonomous roles in the survival, proliferation and/or differentiation of iris, ciliary body and cornea cells may not be discarded.

Complex integration and communication between cell populations derived from the neuroectoderm or neural crest are crucial for proper eye organogenesis. We believe our findings contribute by adding another transcriptional regulator to the already complex set of events required for the coordinated development of multiple eye tissues. Challenges for the future include determining which of the cell populations affected require c-myc function autonomously and understanding the network of transcriptional regulators that regulate or are regulated by c-myc.

## Supporting Information

Figure S1 **Heterozygous inactivation of c-myc in the developing lens partially impairs eye growth.** Representative pictures of hematoxylin & eosin staining in P0 and P30 eyes sections of control (*c-myc^Ctrl^*) (**A**), c-myc heterozygous (*c-myc^Het^*) (**B, C**) and c-myc deficient-lens (*c-myc^Le-Cre^*) (**D, E**). Measurement of eye volume at P30 shows that inactivation of c-myc in developing lens leads to a severe reduction of the eye volume and that the reduction observed is dependent of c-myc dosage (*c-myc^Le-Cre^*; n = 20; *c-myc^Het^*; n = 8; *c-myc^Ctrl^*; n = 12). Error bars indicate SEM. ANOVA test resulted in p<0, 0001 for all comparisons performed.
(TIF)

Figure S2 (**A–B**) Representative pictures of p-Erk staining of control (**A**) and c-myc–deficient (**B**) lens at E13.5. (C**–**D) Representative pictures of ãH2AX staining in control (C) and c-myc deficient (D) lens at P0. (E**–**F) Representative pictures of TUNEL staining of control (E) and c-myc–deficient (F) lens at E12.5. (G**–**H) Representative pictures of TUNEL staining of control (G) and c-myc–deficient (H) lens at P0. Loss of c-myc did not increase apoptotic cell death during embryonic or postnatal lens development. Scale bar: 100 μm. OFZ  =  organelle-free zone.
(TIF)

Table S1 **Primers and Probes Used for Real-Time RT-PCR Analysis.**
(TIF)
