# Supplementary figures and images for: c-myc Regulates Cell Proliferation during Lens Development
Source: PLoS One. 2014 Feb 4;9(2):e87182. doi: 10.1371/journal.pone.0087182 (PMC3913586; doi:10.1371/journal.pone.0087182)

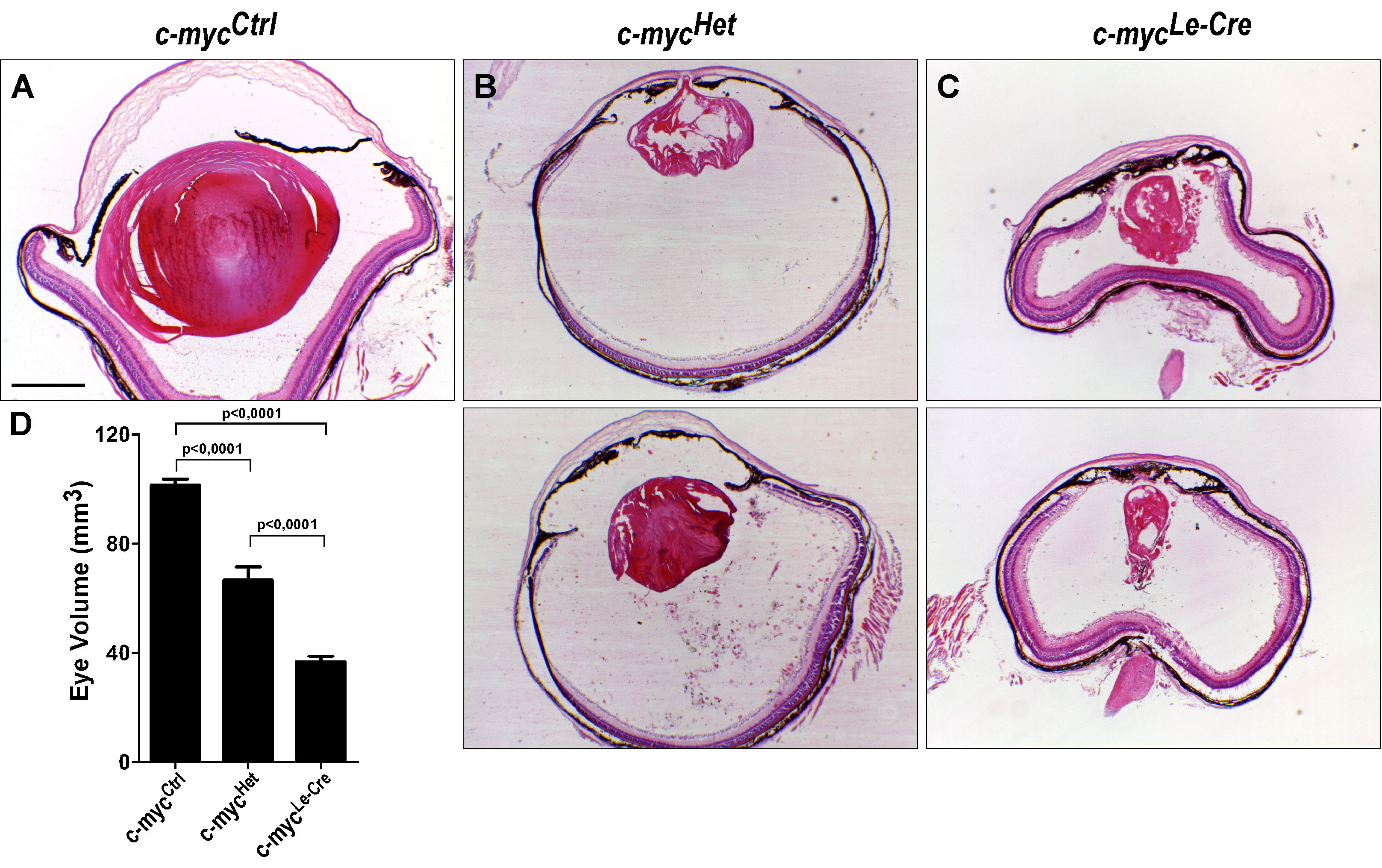

Supplement: Figure S1 — Heterozygous inactivation of c-myc in the developing lens partially impairs eye growth. Representative pictures of hematoxylin & eosin staining in P0 and P30 eyes sections of control (c-mycCtrl) (A), c-myc heterozygous (c-mycHet) (B, C) and c-myc deficient-lens (c-mycLe-Cre) (D, E). Measurement of eye volume at P30 shows that inactivation of c-myc in developing lens leads to a severe reduction of the eye volume and that the reduction observed is dependent of c-myc dosage (c-mycLe-Cre; n = 20; c-mycHet; n = 8; c-mycCtrl; n = 12). Error bars indicate SEM. ANOVA test resulted in p<0, 0001 for all comparisons performed. (TIF) [file pone.0087182.s001.tif]

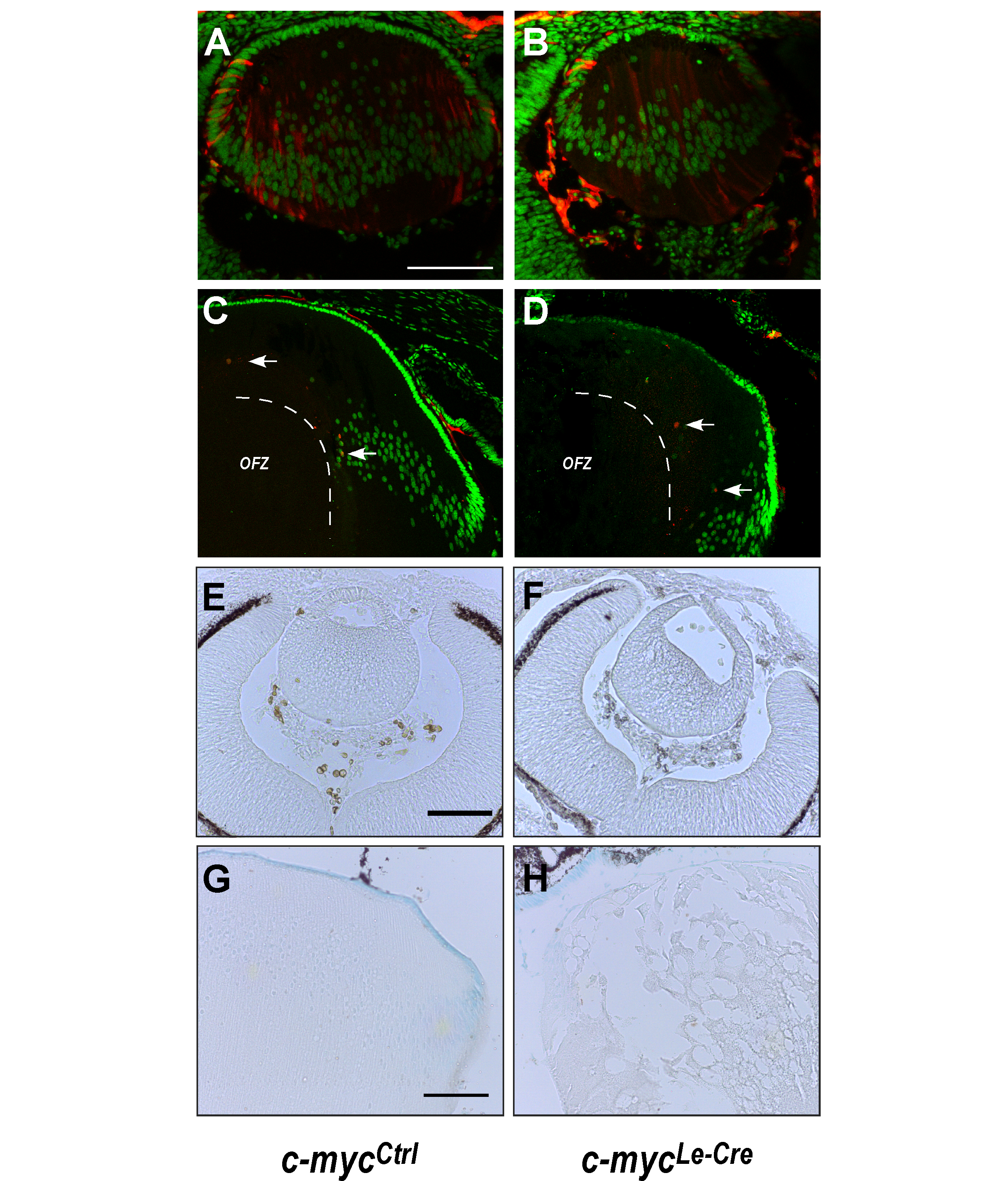

Supplement: Figure S2 — (A–B) Representative pictures of p-Erk staining of control (A) and c-myc–deficient (B) lens at E13.5. (C–D) Representative pictures of ãH2AX staining in control (C) and c-myc deficient (D) lens at P0. (E–F) Representative pictures of TUNEL staining of control (E) and c-myc–deficient (F) lens at E12.5. (G–H) Representative pictures of TUNEL staining of control (G) and c-myc–deficient (H) lens at P0. Loss of c-myc did not increase apoptotic cell death during embryonic or postnatal lens development. Scale bar: 100 μm. OFZ = organelle-free zone. (TIF) [file pone.0087182.s002.tif]

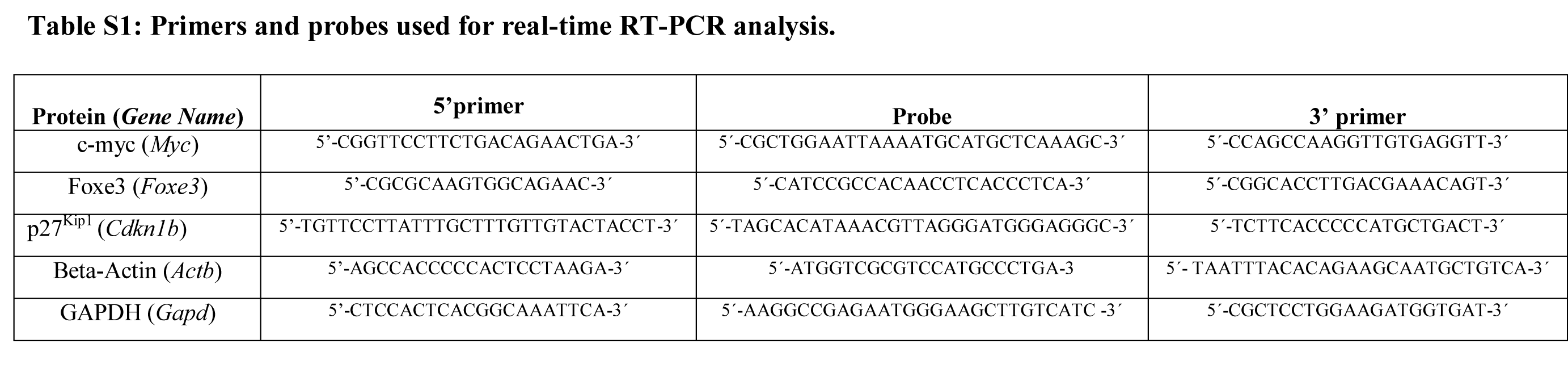

Supplement: Table S1 — Primers and Probes Used for Real-Time RT-PCR Analysis. (TIF) [file pone.0087182.s003.tif]
